# The Source of Respiratory Syncytial Virus Infection In Infants: A Household Cohort Study In Rural Kenya

**DOI:** 10.1093/infdis/jit828

**Published:** 2013-12-23

**Authors:** Patrick K. Munywoki, Dorothy C. Koech, Charles N. Agoti, Clement Lewa, Patricia A. Cane, Graham F. Medley, D. J. Nokes

**Affiliations:** 1 KEMRI–Wellcome Trust Research Programme, Kilifi, Kenya; 2 School of Life Sciences and WIDER, University of Warwick, Coventry, United Kingdom

**Keywords:** RSV, infants, siblings, transmission, households

## Abstract

***Background.*** Respiratory syncytial virus (RSV) vaccine development for direct protection of young infants faces substantial obstacles. Assessing the potential of indirect protection using different strategies, such as targeting older children or mothers, requires knowledge of the source of infection to the infants.

***Methods.*** We undertook a prospective study in rural Kenya. Households with a child born after the preceding RSV epidemic and ≥1 elder sibling were recruited. Nasopharyngeal swab samples were collected every 3–4 days irrespective of symptoms from all household members throughout the RSV season of 2009–2010 and tested for RSV using molecular techniques.

***Results.*** From 451 participants in 44 households a total of 15 396 nasopharyngeal swab samples were samples were collected, representing 86% of planned sampling. RSV was detected in 37 households (84%) and 173 participants (38%) and 28 study infants (64%). The infants acquired infection from within (15 infants; 54%) or outside (9 infants; 32%) the household; in 4 households the source of infant infection was inconclusive. Older children were index case patients for 11 (73%) of the within-household infant infections, and 10 of these 11 children were attending school.

***Conclusion.*** We demonstrate that school-going siblings frequently introduce RSV into households, leading to infection in infants.

(See the editorial commentary by Graham on pages 1679–81.)

Human respiratory syncytial virus (RSV) is a major cause of childhood acute lower respiratory tract infection worldwide [1]. The virus causes seasonal epidemics [2], and approximately 60% of newborn are infected during their first year of life [3]. Concomitantly, the risk of severe RSV associated respiratory disease after infection is highest in early infancy, declining rapidly with increasing age beyond 6 months [4], probably because of physiological maturation of the airways [5]. Vaccine development, in particular for live attenuated virus vaccines, has primarily targeted infants aged <3 months, but no human RSV vaccine has been licensed as yet. There are considerable obstacles confronting the development of vaccines targeting young infants, including immaturity of the immune system and presence of maternal RSV-specific antibodies–both of which are associated with suboptimal vaccine responses, and the balance between immunogenicity and the risk of upper respiratory tract congestion associated with live vaccines [6].

Alternative strategies for RSV vaccination have therefore been proposed [7], including delaying delivery to an older age [8], for which there is empirical support. Live attenuated vaccines have been found safe and immunogenic in seronegative children aged ≥6 months of age [6, 9, 10], subunit RSV vaccines boost protective antibodies in previously infected individuals [11–14], and 40%–60% of RSV-associated community disease that is severe and leads to hospitalization occur in children aged ≥6 months [15–17].

In addition to direct protection of the recipient, vaccination of older age groups may lead to indirect protection of the vulnerable infant by reducing circulation of virus in the population or preventing chains of transmission to the infant [7]. This potential benefit requires knowledge of the source of infection for infants. We conducted a household-based longitudinal study to ascertain from whom infants derive their infection.

## METHODS

### Study Area

The study was undertaken in rural coastal Kenya within the Kilifi Health and Demographic Surveillance System (KHDSS) [18]. One administrative unit in the northern part of the KHDSS, with a population of predominantly subsistence farmers, was selected on the basis of ease of road access and the presence of community health workers assigned to specific households facilitating community entry and engagement. In 2009, the estimated total population in the location was 14 998 persons in 1835 homesteads, each comprising a varying number of households (KHDSS, 2009; unpublished data). The area has 6 public primary schools and 1 nonboarding secondary school. Rains occur twice yearly, usually from April to July and October to December, strongly influencing migration patterns for agricultural purposes. RSV epidemics occur annually. peaking in the first quarter of the each year [16].

### Study Design

A household-based prospective cohort study was set up with a recruitment target of 50 RSV naive infants and their household members. Households (defined in Table 1), identified through KHDSS registers and the local community health workers, were eligible if they included a child born after 1 April 2009, after the end of the 2008–2009 RSV epidemic, and ≥1 older sibling <13 years of age. Enrollment was undertaken before the anticipated start of the 2009–2010 RSV season, and sampling visits were timed to begin and end coincident with the start and finish of the expected RSV season [16]. Households were not enrolled if ≥1 individual refused to participate. Trained field assistants made household visits, collecting deep nasopharyngeal swab (NPS) samples and clinical illness data from all occupants. Community sensitization and identification and recruitment of study households spanned 1 month, followed by a 4-week phase of weekly household visits to collect specimens, thereafter increased to visits every 3–4 days. Households lost to follow-up during the initial phase were replaced. Individuals born into households during the course of the study were recruited. NPS samples were also collected once a week from all field team members.

**Table 1.** Definition of Terms

Term	Definitions
Household	A group of individuals living in the same compound and eating food from the same kitchen
Study infant	The youngest child in the household at the time of recruitment, born after 1 April 2009
RSV season	Periods delimited by weeks in which ≥1 RSV case was identified in hospital surveillance and ≥3 RSV cases were found in any contiguous 3-wk period [16]
Individual episode	Period within which an individual provides specimens that are PCR positive for the same infecting RSV group with ≤14 d separating any 2 positive samples; if an individual had both RSV A and B identified in the first sample of the individual episode, this was coded as a coinfection and counted as 1 individual episode
Household episode	Period within which ≥1 individual episode occurred in members of the same household with no ≥14-d interval without a positive specimen in the household.
Primary case	First individual episode in a household based on sample collection dates; if individual episodes started on the same date in ≥2 members of the same household, they were considered coprimary
Household outbreak	Occurrence of >1 individual episode within a household episode (ie, primary infection spreading to ≥1 other household member)
Visit	Instance in which field staff formally met study participants, at home or at the study clinic, verified by completion of home or clinic visit form; this also includes records of missed appointments (eg, when participants were away from home)

Abbreviations: PCR, polymerase chain reaction; RSV, respiratory syncytial virus.

### Specimen Collection and Handling

The NPS samples were collected using Copan nasopharyngeal flocked swabs (catalog No. 503CS01), following the procedure described elsewhere [19, 20]. On collection, the samples were stored in a cool box with ice packs, delivered to the study clinic for temporary storage at approximately 8°C until transported to KEMRI–Wellcome Trust Research Programme laboratories within 24 hours for processing, and stored frozen at −70°C.

### Laboratory Methods

Viral RNA was extracted from NPS samples using a MagNA Pure LC RNA Isolation Kit—High Performance (Roche Diagnostics) with the MagNA Pure LC Instrument, according to the manufacturer's instructions. Testing for RSV was then performed using an in-house real-time multiplex polymerase chain reaction (PCR) assay [21]. PCR-positive samples were defined as those with a cycle threshold of ≤35. The long ectodomain region of the RSV attachment (G) gene was sequenced as described elsewhere [22].

### Phylogenetic Analysis

The RSV G gene sequences were aligned using the Bioedit program (http://www.mbio.ncsu.edu/bioedit/bioedit), with alignment of RSV groups A and B separately. Comparison of the primary-infant case pairs involved regions 648 and 732 nucleotides long for RSV groups A and B, respectively. Only 1 RSV-positive specimen (with the lowest cycle threshold values) was selected from each pair for sequencing. Phylogenetic trees were constructed using MEGA 5 software [23] with maximum likelihood methods, and branch support was assessed by using 1000 bootstrap iterations. Random sequences (10 for each RSV group, collected from children admitted at the Kilifi District Hospital during a similar period) were included in the trees as references.

### Statistical Analysis

The data were double entered on a custom-made database in Filemaker Pro software (version 9; Filemaker) and analyzed using Stata software (version 11.2; StataCorp). Student *t*, Wilcoxon rank sum, χ^2^, and Fisher exact tests were used as appropriate. The set of terms and their definitions used in the data analysis are given in Table 1. Characteristics were compared between households with or without infant infections. Infected individuals were categorized as study infants (referred to hereafter as *infants*), older children (siblings or cousins aged <15 years), mothers, fathers, or other adults (including aunts, uncles, grandparents, and cousins aged ≥15 years) in the household. Infant infections were categorized as originating from outside the household when the infant was the only primary case patient or the first individual identified as RSV positive in a household outbreak and from within the household if another individual was the first in the household identified as RSV positive. The origin was deemed inconclusive when the infant and another household member were concurrently first to be identified as RSV positive (ie, coprimary) in a household outbreak. For within-household spread and coprimary cases, RSV attachment (G) gene nucleotide sequences were compared for the primary case(s) and infant infections.

### Ethical Clearance

The study was approved by the Kenyan National Ethical Review Committee and the University of Warwick's Biomedical Research Ethical Committee in the United Kingdom. Individual written informed consent was obtained from all study participants aged ≥18 years. For those <18 years old, written consent was obtained from the parent or guardian.

## RESULTS

### Households Recruited

**Figure 1.**
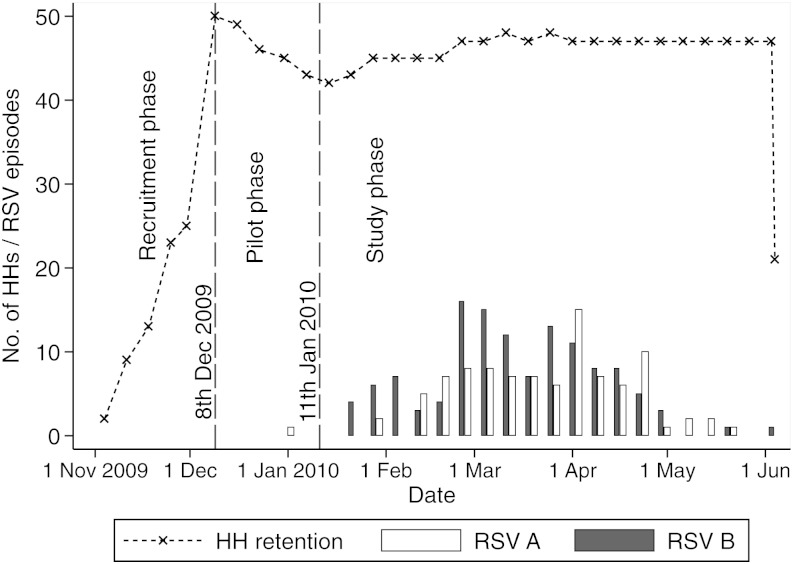
Number of households participating and individual episodes of respiratory syncytial virus (RSV) A and B infection detected during the follow-up period (weekly delimited data) in 2009–2010.

Household recruitment started on 4 November 2009 and reached 50 by 8 December 2009, when regular specimen sampling began. Nine households withdrew during this period, with 2 replacements. The study was fully in operation, including sampling every 3–4 days, by 11 January 2010, the official study start date. Subsequently, another 4 households were lost to follow-up, and 8 replacement households were recruited, with the last recruitment taking place on 5 March and the last withdrawal on 7 March 2010. The study closed on 4 June 2010, after 24 weeks of follow-up, Figure 1. Overall, 60 households (596 participants) were recruited, and 13 households (103 participants) were lost to follow-up. Of those lost to follow-up, 6 households were never sampled (3 declined the initial sampling, 2 became ineligible because the parents separated, and 1 became ineligible because of migration); 6 households withdrew in the course of the study, with the members citing dislike or fear of the frequent nasopharyngeal swabbing; and 1 household out-migrated after sampling started. In addition, 3 households were excluded from this analysis because the study infant was infrequently sampled during the peak months of RSV infection. All subsequent analyses thus include data from 44 households.

Household characteristics are summarized in Table 2. All study infants were born after 1 April 2009, with a median age at recruitment of 4.4 months (interquartile range, 2.4–6.6 months; range, 13 days to 9.9 months). Twenty-one infants (47.7%) were male.

**Table 2.** Baseline Characteristics of Study Infants and Their Households

Characteristic	Statistic
Study infants (n = 44)	
Male sex, No. (%)	21 (47.7)
Age at recruitment, median (IQR), mo	4.2 (2.4–6.4)
Other household members (n = 407)	
Male sex, No. (%)	186 (45.7)
In school, No. (%)	154 (37.8)
Age at recruitment, median (IQR), y	12.6 (6.6–26.5)
Frequency distribution of members by age at recruitment, No. (%)	
0 to <1 y	6 (1.5)
1–4 y	74 (18.2)
5–14 y	153 (37.6)
15–39 y	133 (32.7)
≥40 y	41 (10.1)
Household members, median (IQR), No.	
Total	8 (6–11.5)
Older children (aged 1–14 y)	4 (3–6)
School-going children	4 (3–6)
Members living in same building unit as study infant	5 (3–6)
Mothers with no formal education, No. (%)	10/43 (23.3)
Duration of household follow-up, median (IQR), wk	24.8 (23.6–25.3)
Frequency distribution in RSV episodes per household, No. (%)	
0	7 (15.9)
1	16 (36.4)
2	11 (25.0)
3	8 (18.2)
≥4	2 (4.5)

Abbreviations: IQR, interquartile range; RSV, respiratory syncytial virus.

### Household Visits and Sample Collections

Based on our sampling regimen, a total of 17 985 collected NPS samples were expected during the study phase. In practice, we contacted 16 434 participants (91.4%), obtaining 15 396 NPS samples (85.6% of expected). The proportion of expected NPS samples collected was highest in study infants (approximately 100%) and lowest among other adults in households (64.3%; Table 3). A median of 39 NPS samples were collected per individual (interquartile range, 30–42 samples).

**Table 3.** Visits and NPS Sample Collections From 451 Participants During Study Phase, Stratified by Relation of Individuals to Study Infant

Study Participants^a^	Individual Visits, No. (%)			Clinic Visits, No.	NPS Sample Collections, No. (%)		
	All^b^	At Home	Away		Expected^c^	Collected^d^	When Symptomatic^e^
Study infants (n = 44)	1846	1764 (95.6)	82 (4.4)	92	1752	1763 (100.6)	912 (52.1)
Siblings (n = 157)	6453	6109 (94.7)	344 (5.3)	81	6224	5871 (94.3)	1710 (27.5)
Cousins (n = 105)	4197	3662 (87.3)	535 (12.7)	58	4202	3378 (80.4)	749 (17.8)
Mothers (n = 43)	1792	1708 (95.3)	84 (4.7)	45	1716	1695 (98.8)	162 (9.4)
Fathers (n = 31)	1239	1024 (82.6)	215 (17.4)	7	1224	845 (69.0)	51 (4.2)
Other adults (n = 71)	2741	2167 (79.1)	574 (20.9)	45	2867	1844 (64.3)	178 (6.2)
Overall (n = 451)	18 268	16 434 (90.0)	1834 (10.0)	328	17 985	15 396 (85.6)	3762 (20.9)

Abbreviations: NPS, nasopharyngeal swab.

^a^ Household members stratified by relation to study infant.

^b^ Includes all instances in which field workers formally visited study participants at home, verified by completion of the home visit form and including records of when the participant was away from home.

^c^ Total number of NPS sample collections expected if samples were collected twice a week, discounting periods when household members were reported to be away from the household for >3 consecutive days.

^d^ Additional samples were collected during clinic visits when participants were symptomatic.

^e^ NPS samples collected when participant had symptoms of acute respiratory illness defined by presence of ≥1 of the following symptoms: cough, blocked nose/nasal discharge, or difficult in breathing.

### RSV Infections in Households

Overall, 84.1% of households (37 of 44) and 38.4% of individual participants (173 of 451) had ≥1 episode of RSV infection. A total of 73 separate introductions into households were identified, with 32 (43.8%) resulting in a household outbreak. The 32 outbreaks were identified in 27 households: 1 outbreak in 23 households (8 with RSV group A, 14 with RSV group B, and 2 with a coinfection of both) and 2 outbreaks in 4 households (2 with RSV group A followed by group B infection and 2 with consecutive group B infections). The study infant was infected in 28 (87.5%) of the household outbreaks. Households with RSV spread among the members had a higher mean number of older children than those without RSV infection, although the difference was not statistically significant (mean, 6.0 [95% confidence interval, 4.4–7.6] vs 3.3 [1.8–4.8]; *P* = .08; Supplementary Table 1).

### Source of the Infant Infections

**Figure 2.**
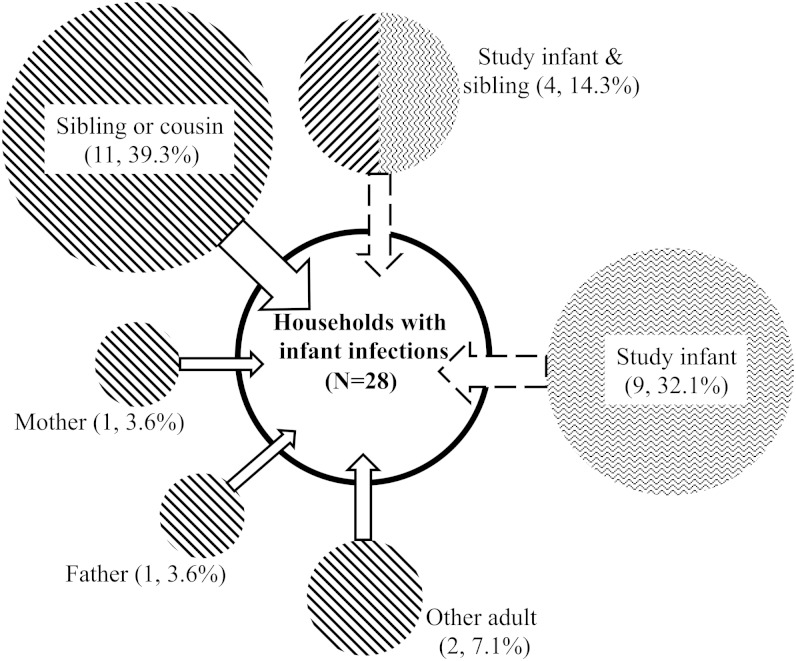
Distribution of primary cases for the 28 household episodes linked with the study infant infection in rural Kenya. Only the first household episodes/outbreaks involving the study infants are shown. The diagonal and zigzag lines shading the circles indicate outside- and within-household acquisition of the infant infections, respectively, and the area of the circle is in proportion to the number of cases in each category.

Of the 73 separate household episodes identified, 37 (50.7%) resulted in the infection of the study infants. Overall, 28 of the 44 study infants (63.6%) were infected: 21 had 1 episode, 5 had 2, and 2 had 3 episodes. The 28 infant infections that were first episodes were associated with an outbreak in 24 households (85.7%). Subsequent results for the infant infections are limited to these first episodes. The results for all infant episodes are shown in Supplementary Figure 1. Based on the temporal pattern of infections in each household, 15 (53.6%) of the 28 study infant infections were acquired through transmission within households, and 9 (32.1%) were acquired from outside the household. The source of infant infections in the remaining 4 households (14.3%) was inconclusive because the household episodes had a sibling and the infant as coprimary case patients (Figure 2).

**Figure 3.**
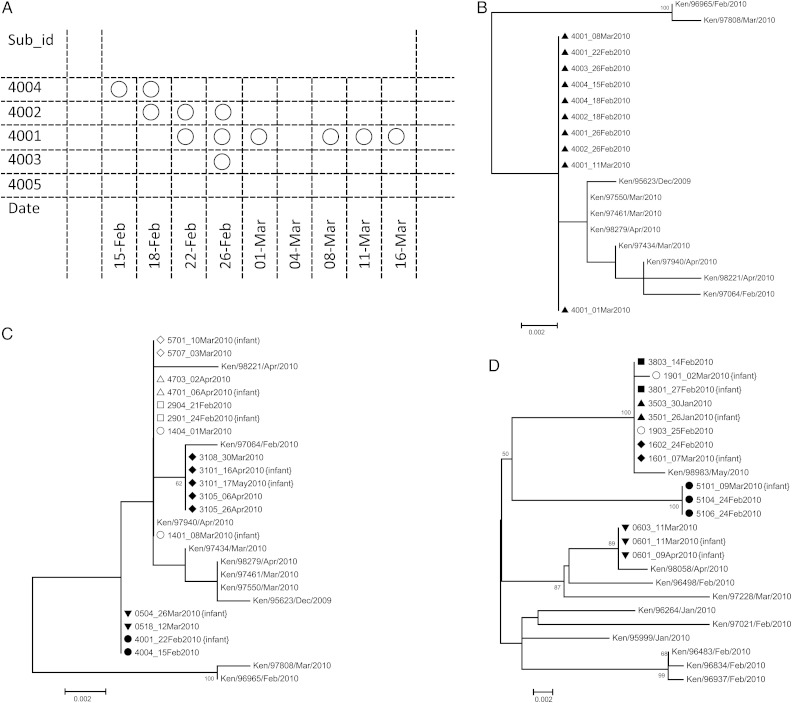
*A,* Temporal occurrence of respiratory syncytial virus (RSV)–positive samples in a household of 5 members (household 40). Each box represent a sample collected; each circle, an RSV-positive sample. RSV was introduced by subject 4004. *B*, An Maximum Likelihood phylogenetic tree of 10 of 12 samples from household 40 together with 10 RSV A reference sequences from Kilifi District Hospital (KDH). All samples from household 40 (preceded by triangles on tree) had identical sequences in the G region sequenced. *C*, Phylogenetic relationship of the G similarity of study infant–primary case pairs for RSV A. Samples from the same household are preceded by the same symbol (filled or open). Taxon naming for the household samples follows household number with individual number and date of sampling (eg, 4703_02April2010 represents household 47, individual 3, and sample collected on 2 April 2010). The KDH RSV A reference sequences are as in *B. D*, Same as *C,* but for RSV B. An example RSV B pairing is 3803_14Feb2010 and the later infant-infecting virus sequence 3801_27Feb2010. Ten random samples from the KDH inpatient studies are included for reference, as in *B* and *C*.

Samples were successfully sequenced for the RSV G gene region in 10 of 15 primary case patient and study infant pairs, with all pairs showing complete homology of nucleotide sequence (Figure 3). In the remaining 5 pairs in which sequencing failed, ≥1 sample from each pair had a high cycle threshold value (>30), a proxy for low virus load, based on the real-time PCR data. Sequences from the coprimary cases were identical in 3 of the 4 sibling-infant pairs. The primary case patients for the 15 study infants who seemed to have acquired the infection from within the household included 11 older children, 1 mother, 1 father, and 2 other adults.

A similar distribution of primary cases was seen for analysis of all household episodes and outbreaks (Supplementary Table 2). Ten of the 11 older children who were primary case patients were attending school, and 1, an adult (uncle to the study infant, aged 17 years), was in secondary school. The mean age at recruitment of these older children was 6.9 years (range, 2.3–11.5 years). In the 4 households in which siblings were coprimary case patients with infants, 2 siblings were attending school. There were no significant differences in individual or household characteristics between infected and uninfected infants (Table 4). None of the field team members had RSV infection detected during the study period.

**Table 4.** Characteristics of Households With or Without Respiratory Syncytial Virus Infections in Study Infants^a^

Household Characteristic	No Infection in Infants (n = 16)	Infection in Infants (n = 28)	*P* Value
Household members, No.	8.0 (7.0–9.0)	9.0 (6.0–14.5)	.44
Age of members, mean (95% CI), y	16.0 (14.2–17.8)	15.8 (14.3–17.2)	.77
Age of study infant, mo	4.3 (3.1–5.5)	4.7 (3.7–5.7)	.63
Older children, No.	4.0 (3.0–5.0)	5.0 (3.0–7.5)	.23
Male household members, %	50.0 (40.0–57.1)	41.4 (35.8–57.7)	.44
School-going children, No.	4.0 (2.0–4.0)	4.0 (3.0–7.0)	.24
Members living in same building unit with study infant, No.	6.0 (4.5–6.0)	4.5 (3.0–6.5)	.23

Abbreviation: CI, confidence interval; RSV, respiratory syncytial virus.

^a^ Data represent medians (interquartile ranges) except where otherwise indicated.

## DISCUSSION

We report on a study designed to determine the source of RSV infection in infants during their first epidemic, as those who are most vulnerable to disease from RSV infection. The aim was to establish the proportion of infants infected from a source within the immediate household and, in such households, to determine which member was responsible for introducing RSV into the household. Data from rural coastal Kenya are presented for 44 households, with 451 members recruited and followed up actively for NPS sample collection every 3–4 days regardless of symptoms during a full RSV epidemic. Sensitive diagnosis of infection was achieved by using a real-time PCR assay. Through a lengthy process of community engagement and trust building, it was possible to achieve high household retention and sample collection during the 6 months of the study. RSV infection was detected in 84.1% of the households and 38.4% of the participants. Both known RSV subtypes (A and B) circulated in the community during the study period, which helped distinguish infection episodes and transmission events.

The study infant was infected at least once in 28 of the 44 households, with about a third (32.1%) acquiring the infection from outside the household and about half (53.6%) from within the household. In the remaining (14.3%) households, the source of infant infection was undetermined because the infant and sibling were coprimary case patients. Children attending school were important in the within-household transmission, comprising 67% of the total (ie, 10 of 15) and 90% of the childhood case patients (ie, 10 of 11). Mothers or fathers were less likely to be the primary case patients. There were no statistically significant differences between the individual or household characteristics of infected and uninfected infants, suggesting that differences between households are based largely on chance or factors not observed in the current study. In addition, the distribution of the primary cases was similar for isolated household episodes (ie, episodes with no household spread) and outbreaks of RSV, indicating that whether or not the infection spreads is determined not by who brings the infection into the household but maybe by chance or by an unobserved characteristic of the household, such as whether an outbreak was experienced in the previous year(s).

Only 1 study in the past has looked at the spread of RSV within households in detail [24]. Hall and colleagues recruited 36 US families with young infants, collecting nose or throat samples twice a week regardless of symptoms during 2 months of an RSV season and assessing infection using viral culture [24]. RSV was detected in 16 (44.4%) of the families and 21.9% of the 188 members, lower rates than in the current study (84.1% and 38.4%, respectively). Based only on temporal occurrence of infections, RSV seemed to be introduced by siblings in 8 (50.0%) of 16 infected families; by other household members, including parents, in 3 families (18.8%); and with involvement of an infant in 5 (31.3%; coprimary cases). The corresponding statistics for the 32 household outbreaks recorded in our study were 16 (50.0%), 5 (15.6%), and 11 (34.4%). As in our study, these findings highlight the importance of older children in introducing RSV into households with young infants. Our study also shows that children with daily contact with many other children, particularly in schools, are important in bringing RSV infections into the households.

Other, less comparable studies have implicated siblings [25–29] and mothers [30] as source of infections in the family for young infants. However, none of these studies [25–30] were designed specifically to identify the source of infant RSV infections in families. Our current study and the family study by Hall et al in Rochester, New York [24] were designed to identify transmission chains by sampling frequently and irrespective of symptoms. However, because methods have changed since the US study [24], our study has the advantages of increased sensitivity in case detection with PCR and of sequence data support for observed primary-infant infection pairs. Moreover, the US study is likely to have missed infections early in the RSV epidemic owing to sampling delay and suffered a high proportion of coprimary cases, restricting the ability to identify relative individual contributions as infection sources within families. Nevertheless, the consistency of results, given the contrasting locations and different times, suggest that the important role of a school-age sibling in bringing infection into the household is not unique to a particular social and demographic setting.

Our results support the notion that preventing infection in school-going children could indirectly reduce RSV infection in infants. However, an assessment of the impact of such an intervention requires consideration of the competing risks; for example, somebody other than the vaccinated child might introduce infection or the infant might be infected in the community if not the household. However, targeted sibling immunization would nonetheless result in fewer susceptible individuals within the household, which would reduce spread and provide indirect infant protection, and any reduction in rate of infection to infants will translate into a delay in infection to an older age, reducing the risk of disease.

These results also point to schoolchildren as the “core group” for RSV. Consequently, universal immunization of children (regardless of sibling status) could reduce circulation of RSV in the community, especially in typical rural African communities with many children like the one we studied, further reducing the risk of infant infection. However, such an intervention is more likely to interrupt the transmission dynamics of RSV and may change the seasonality and the age distribution of susceptibility and infection. We are currently conducting modeling studies to address these issues by further clarifying the details of transmission within the household and the impact of mass immunization.

The current study also has some limitations. First, generalizability of the results is limited in that we specifically selected households with an older child, and the households were few and within a tight geographic area populated by rural farmers. It would be important to identify the source of infant infections in households without older children. However, only 19.6% of the infants in the KHDSS live in households without an older child in their compound. Second, the frequency of sampling might have missed short-duration RSV shedding (if <3 days), including primary cases, despite the intensive sampling. For some instances in which the infant is the primary or coprimary case patient, the index case patient may have gone unidentified. This risk is larger than the alternative (wherein the infant was actually the first case patient but not identified as such by us), because sample collection in infants was thorough and infants were mainly infected in the first few days after infection was introduced into household (Supplementary Figure 1 in the Supplementary material). We therefore believe that our estimate of the importance of within-household spread as a source of infant infections is an underestimate. Third, given that it is more difficult to diagnose RSV in adults than in children [31], the role of parents or grandparents in household RSV may have been underestimated. However, the use of sensitive real-time PCR will have mitigated problem this to a significant degree. The serological identification of cases may have reduced misclassification of infection but would not have helped in the resolution of transmission chains. Further work is planned using full-genome sequencing in positive samples to identify molecular fingerprints that might help delineate the chains of RSV spread within households.

In conclusion, we demonstrate that in this rural Kenyan location, for a high proportion of infant RSV infections, the source is from the household and predominantly introduced by an elder school-going sibling. Thus, there is potential for targeted immunization of older siblings of naive infants, or universal vaccination of older infants and children, to reduce the spread of RSV and the risk of infection entering households and to delay first infection in infants to an older age at which associated disease is less of a risk.

## Supplementary Data

Supplementary materials are available at *The Journal of Infectious Diseases* online (http://jid.oxfordjournals.org/). Supplementary materials consist of data provided by the author that are published to benefit the reader. The posted materials are not copyedited. The contents of all supplementary data are the sole responsibility of the authors. Questions or messages regarding errors should be addressed to the author.

Supplementary Data
