# Supplementary material for: The Source of Respiratory Syncytial Virus Infection In Infants: A Household Cohort Study In Rural Kenya
Source: J Infect Dis. 2013 Dec 23;209(11):1685–92. doi: 10.1093/infdis/jit828 (PMC4017365; doi:10.1093/infdis/jit828)
Supplement: Supplementary Data [file supp_209_11_1685__index.html]

The Source of Respiratory Syncytial Virus Infection In Infants: A Household Cohort Study In Rural Kenya — The Source of Respiratory Syncytial Virus Infection In Infants: A Household Cohort Study In Rural Kenya — Supplementary Data 

# The Source of Respiratory Syncytial Virus Infection In Infants: A Household Cohort Study In Rural Kenya

## Supplementary Data

Supplementary Data

**Files in this Data Supplement:**

- Supplementary Data - Docx file
