# Supplementary material for: The Source of Respiratory Syncytial Virus Infection In Infants: A Household Cohort Study In Rural Kenya
Source: J Infect Dis. 2013 Dec 23;209(11):1685–92. doi: 10.1093/infdis/jit828 (PMC4017365; doi:10.1093/infdis/jit828)
Supplement: Supplementary Data [file supp_jit828_jit828supp.docx]

**The source of respiratory syncytial virus infection in infants: a household cohort study in rural Kenya**

Patrick K. Munywoki^1*^; Dorothy Koech^1^; Charles N. Agoti^1^; Clement Lewa^1^; Patricia A. Cane^2^; Graham F. Medley^2^; D. J. Nokes^1, 2^

Institutional affiliations

1. KEMRI - Wellcome Trust Research Programme, Kilifi, Kenya
2. School of Life Sciences and WIDER, University of Warwick, Coventry, UK

*Corresponding Author

Patrick Kiio Munywoki, KEMRI-Centre for Geographic Medicine Research - Coast, Hospital Road, P.O. Box 230, Kilifi, Kenya; Tel +254 41 522063; email, [pmunywoki@kemri-wellcome.org](mailto:pmunywoki@kemri-wellcome.org)

**Supplementary Material**

**Supplementary Results**

*Primary cases of all the household episodes*

From the 73 household episodes, a total of 71 different individuals were primary cases among which 31 (43.7%) individuals were associated with the 32 household outbreaks detected, Table S3. Primary cases of all household episodes were study infants, 15 (20.5%); older children, 36 (49.3%); study infants and older child, 4 (5.5%); mothers, 9 (12.3%); fathers, 4 (5.5%); and other household members, 5 (6.8%). The corresponding distribution of the primary cases for household outbreaks was 7 (21.9%), 16 (50.0%), 4 (12.5%), 2 (6.3%), 1 (3.1%), and 2 (6.3%).

**Supplementary Tables**

Table S1: Characteristics of households with and without RSV infection episodes

| ***Characteristics^1^*** | ***Uninfected HHs***  ***(n=7)*** | ***Infected HHs with no virus spread***  ***(n=10)*** | ***Infected HHs with virus spread (n=27)^2^*** |
| --- | --- | --- | --- |
| Household size | 8 (5 – 9) | 8 (7 – 9) | 10 (6 –15.5) |
| Mean age in years per HH | 16.3 (13.4 – 19.2) | 15.5 (13.0 –19.0) | 14.6 (13.5 –17.5) |
| No. of older children in HH | 3 (2 – 4) | 4 (4 –5) | 5 (3 – 8.5) |
| Male: female ratio | 1.7 (1.1) | 1.0 (0.5) | 1.5 (1.2) |
| No. of school going children | 3 (2 – 4) | 4 (3 – 5) | 4.5 (3 – 7.5) |
| No. of children living in same house per HH | 5 (3 – 6) | 6 (5 – 7) | 4 (2.5 – 6) |
| No. of children sleeping in same room per HH | 3 (2 – 4) | 3 (3 – 5) | 3 (2 – 3.5) |
| No. of children sleeping in same bed per HH | 2 (2 – 2) | 2 (2 – 2) | 2 (2 – 2) |

Key: HH, household; 1, Reported statistics are the median and interquatile range except for age which is mean and 95% confidence interval; 2, limiting this column to the 24 households with outbreaks involving the study infant does not significantly change the statistics shown. There were no statistically significant differences between the infected households compared to uninfected households in the observed characteristics

Table S2: Characteristics of the primary cases of the household episodes and outbreaks

|  | **HH episodes^1^**  **(N=41)** | | **HH outbreaks^2^**  **(N=32)** | | **HH outbreaks with infant infection^3^ (N=28)** | |
| --- | --- | --- | --- | --- | --- | --- |
| *Characteristic* | *n* | *%* | *n* | *%* | *n* | *%* |
| *Relation to the study infant* |  |  |  |  |  |  |
| Self | 8 | 19.5 | 7 | 21.9 | 7 | 25.0 |
| Self and sibling^4^ | - | - | 4 | 12.5 | 4 | 14.3 |
| Sibling or cousin^5^ | 20 | 48.8 | 16 | 50.0 | 12 | 42.9 |
| Mother | 7 | 17.1 | 2 | 6.3 | 2 | 7.1 |
| Father | 3 | 7.3 | 1 | 3.1 | 1 | 3.6 |
| Others | 3 | 7.3 | 2 | 6.3 | 2 | 7.1 |
| Age groups in years |  |  |  |  |  |  |
| <1y | 11 | 26.8 | 10 | 31.3 | 10 | 35.7 |
| 1-4y | 5 | 12.2 | 6 | 18.8 | 6 | 21.4 |
| 5-14y | 13 | 31.7 | 11 | 34.4 | 7 | 25.0 |
| 15 – 39y | 10 | 24.4 | 4 | 12.5 | 4 | 14.3 |
| 40+y | 2 | 4.9 | 1 | 3.1 | 1 | 3.6 |
| Male gender | 18 | 43.9 | 17 | 53.1 | 15 | 53.6 |
| Older children | 18 | 43.9 | 17 | 53.1 | 13 | 46.4 |
| Older children in school | 14 | 82.5 | 15 | 88.2 | 15 | 84.6 |

Key: HH – household; 1, limited to household episodes with no spread within the household; 2, includes all household outbreaks, five households had two outbreaks detected; 3, includes household outbreaks associated with the study infant infection, four households had two outbreaks; 4, co-primary cases of the study infant (self) and siblings; 5, either sibling or cousins to the study infant

**Supplementary Figures**


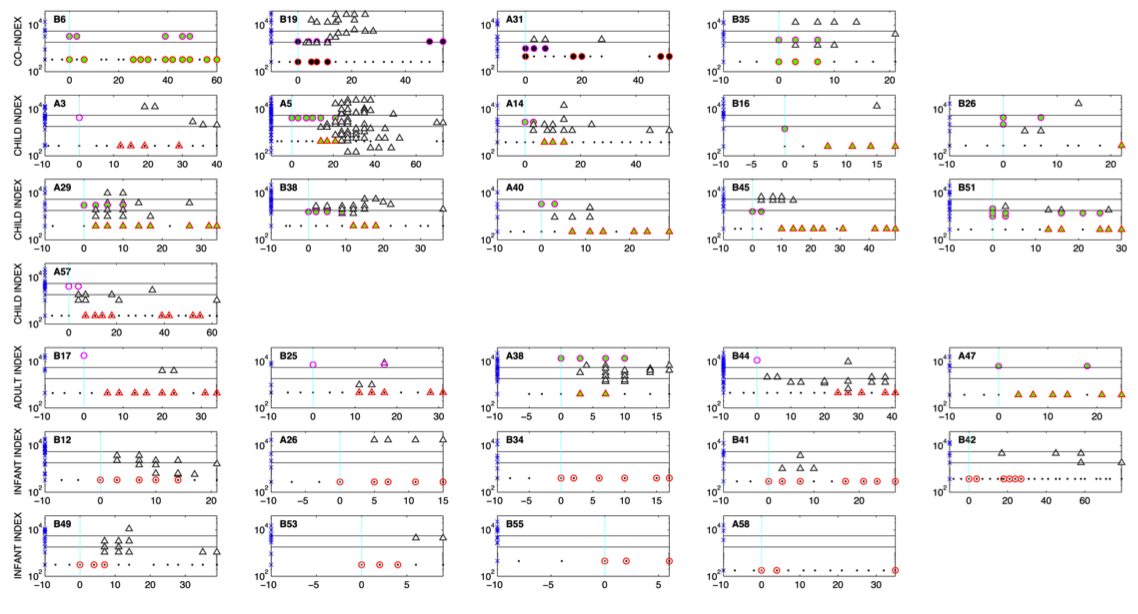


Figure S1: RSV transmissions patterns in the 28 households with infant infections. Each panel shows household episode(s) stratified by RSV group i.e. B6 is RSV group B episodes in household number 6. The RSV group (A or B) and household number are in the top left of each panel. The vertical axis is the log_10_ (age) and each individual in the household is shown with a blue cross. Two horizontal lines show the ages of 5yrs and 15yrs. The horizontal axis shows the number days relative to the first (primary) case. Each symbol represents a NPS. For the study infant each NPS is marked with a black dot, but for other members, negative samples are omitted for clarity. Circles show the index case and the top row show households with co-primary cases. Non-primary cases are shown with triangles. Filled symbols show that the virus from this infection was successfully sequenced. If the colour is black, then the viruses are different, and if the colour is green, the viruses are the same. The black dots represent NPS collections from the study infant.
